# Localised Leishmaniasis of Oral Mucosa: Report of an Unusual Clinicopathological Entity

**DOI:** 10.1155/2014/753149

**Published:** 2014-09-29

**Authors:** Deepak Passi, Sarang Sharma, Shubharanjan Dutta, Chandan Gupta

**Affiliations:** ^1^Department of Oral & Maxillofacial Surgery, E.S.I.C Dental College & Hospital, Rohini, Delhi 110089, India; ^2^Department of Conservative Dentistry & Endodontics, E.S.I.C Dental College & Hospital, Rohini, Delhi 110089, India; ^3^Department of Oral & Maxillofacial Surgery, Vyas Dental College & Hospital, Jodhpur, Rajasthan 342005, India; ^4^Department of Oral & Maxillofacial Surgery, Faculty of Dental Sciences, King George's Medical University, Lucknow, Uttar Pradesh 226003, India

## Abstract

The term leishmaniasis comprises of a group of diseases caused by different species of a protozoan called Leishmania. There are three main clinical forms of leishmaniasis: visceral leishmaniasis, cutaneous leishmaniasis, and mucocutaneous leishmaniasis. Exclusive involvement of the mucosa is very rare. We present a case of mucosal leishmaniasis located in the oral cavity. The only manifestation of leishmaniasis disease in the described case was the appearance of an oral lesion. Treatment was provided in the form of antimoniates (oral miltefosine and systemic sodium stibogluconate). A review of literature is made on the subject.

## 1. Introduction

The term leishmaniasis comprises of a group of diseases caused by a species of protozoan called* leishmania*, transmitted by female sandflies of the genera* Phlebotomus and Lutzomyia*. It belongs to the family Trypanosomatidae, genus:* Leishmania*. Synonyms for leishmaniasis are kala azar, meaning black fever in India, and espundia (mucocutaneous form). Leishmaniasis is found worldwide and is considered to be endemic in 88 countries [1, 2]. The incidence of leishmaniasis as an opportunistic disease has increased in recent years because of the growing number of patients with immune depression secondary to chronic illnesses, neoplasms, immunosuppressive treatments, transplants, and HIV infection [1, 3]. Its prevalence is 12 million infected individuals worldwide with a global incidence of 1.5–2 million new cases per year. Leishmaniasis is responsible for approximately 70,000 to 80,000 deaths a year [4].

It occurs in three clinical forms—*cutaneous, mucocutaneous, and visceral* [3].

In the literature two different versions are discussed, that is, Old World cutaneous leishmaniasis (OWCL) and New World cutaneous leishmaniasis (NWCL).* Cutaneous leishmaniasis (CL)* causes skin sores and chronic ulcers. It is generally self-limiting but can be a chronic and progressive disease in a proportion of cases.* Mucocutaneous leishmaniasis* caused by* Leishmania* species in Africa and America affects the nasal, oral, and pharyngeal mucosa, producing a disabling and mutilating disease.* Visceral leishmaniasis* affects the spleen, liver, bone marrow, and lymph nodes, producing fever and anaemia. It is usually fatal if untreated.

Leishmaniasis afflicts males and females in the ratio of 2 : 1. In immunocompromised patients, oral mucosa is the second most frequently affected site of the head and neck region. In the oral cavity, tongue is the most affected site. Mucocutaneous disease is due to extension of local skin disease into the mucosal tissue via direct extension, bloodstream, or lymphatics. Lesions mainly involve the oral and nasal mucosa and occasionally the laryngeal and pharyngeal mucosa. If not recognized and adequately treated, it may disfigure the patient because of chronic local destruction of the tissues of the nose, pharynx, and palate. Generally, mucosal involvement develops 1–5 years after the healing of cutaneous leishmaniasis, though in some cases mucosal and skin lesions may coincide. Approximately 90% of patients present with prior cutaneous scar. Occasionally, mucosal lesions may also appear in patients with no previous skin lesions [5]. Exclusive involvement of the mucosa is very rare. We present a case of leishmaniasis where the only manifestation of disease in the reported case was the appearance of oral lesions without cutaneous involvement.

## 2. Case Report

**Figure 1:**
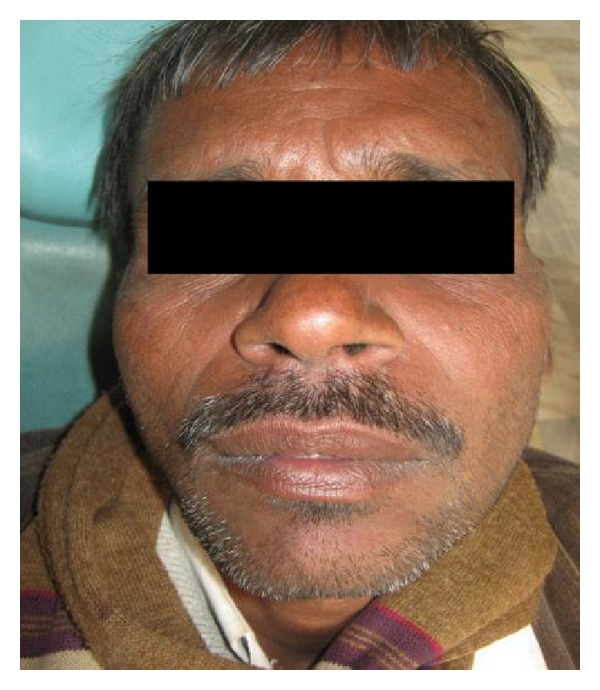
Profile view showing no cutaneous lesion.

**Figure 2:**
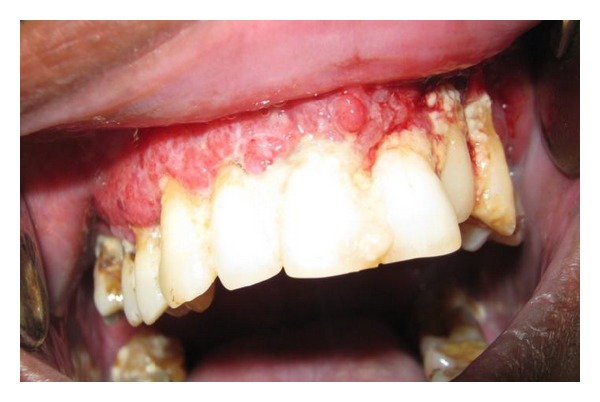
Involvement of labial mucosa and gingival hypertrophy with bleeding.

**Figure 3:**
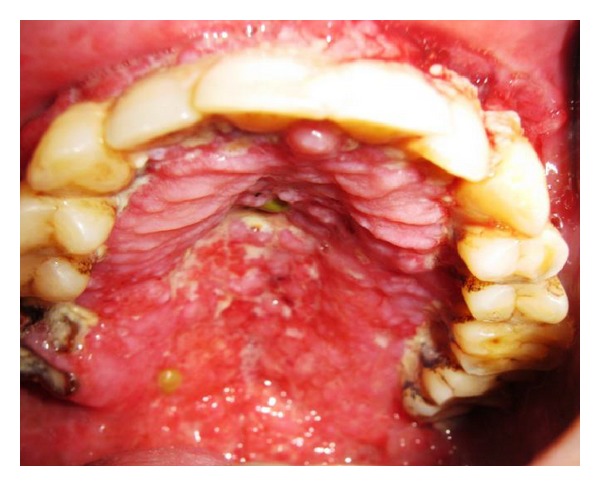
Involvement of hard and soft palate (cobblestone appearance of mucosa of the hard palate).

**Figure 4:**
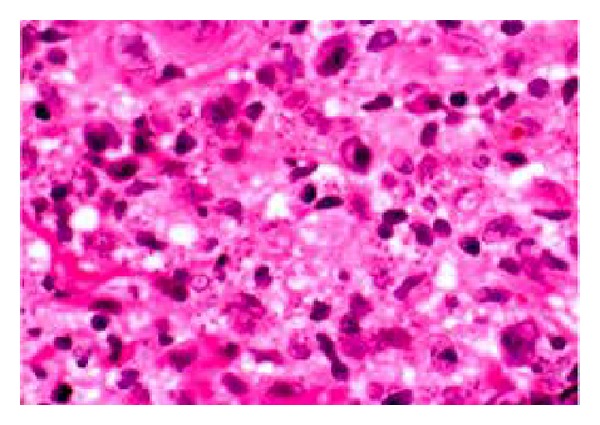
Histology of the oral lesion showing macrophages containing numerous Leishman bodies.

**Figure 5:**
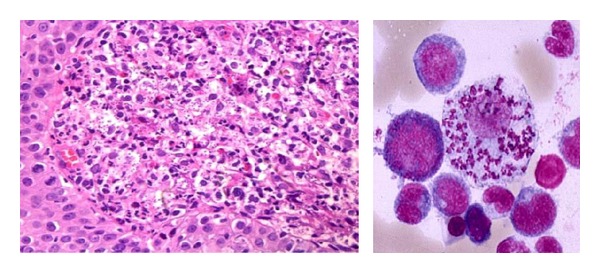
Giemsa staining shows numerous amastigotes with round nucleus.

A 51-year-old male reported to the Department of Oral and Maxillofacial surgery, King George's Medical University, India, with chief complaint of bleeding gums and ulceration of mouth leading to difficulty in mastication and swallowing (Figures 1, 2, and 3). Past medical history was suggestive of fever 1 month back with lymphadenopathy. Personal history revealed the patient to be a driver by profession with habit of tobacco chewing and smoking since last 5 years. On general examination, there were no lesions/scars on skin of face, trunks, and extremities and also there was* no history of previous cutaneous lesion*. On intraoral examination, there were present tender, diffuse, and multiple ulcerated nodules (less than 1 cm) on the hard and soft palate with surface hoarseness. Associated maxillary labial gingiva presented with cobblestone appearance, had marked erythema and was excrescent with granular inflammation. Oral hygiene was poor and lesions bled on exploration.* In our case, there was exclusive involvement of oral mucosa of maxillary labial/buccal surface and palate without cutaneous involvement*. Neck examination revealed bilaterally enlarged and tender submandibular lymph nodes. ENT opinion was taken for dysphagia and was given symptomatic treatment. Patient was investigated with biopsy of the lesion from two different sites. One tissue sample was taken from maxillary labial gingiva and the second from lateral margin of palate from the edge of ulcer. Hematoxylin-eosin (H&E) and Giemsa staining of the biopsy confirmed the diagnosis of leishmaniasis (Figures 4 and 5). Patient's routine blood investigation revealed leukocytosis. Viral markers, that is,* HIV*,* HBsAg*, and* HCV*,* were nonreactive*.

**Figure 6:**
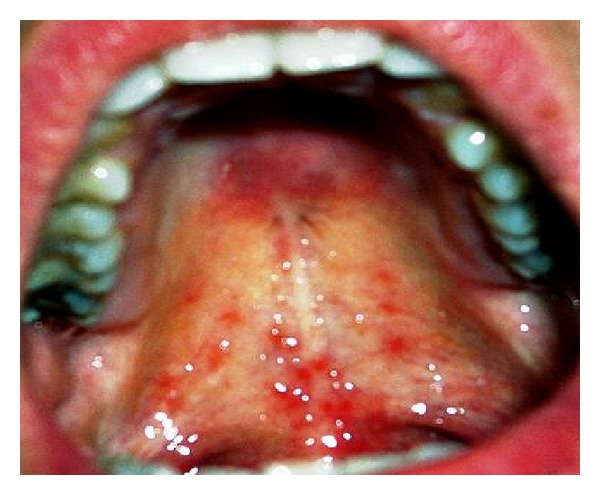
Healing of lesion.

Patient was treated with oral miltefosine and systemic sodium stibogluconate: 100 mg miltefosine daily as one capsule (50 mg) in the morning and one capsule (50 mg) in the evening, after meals for 28 days, and 20 mg sodium stibogluconate (0.2 mL Pentostam) per kg bodyweight daily intravenously for 30 days. Patient was regularly monitored through out with blood investigations and ECG during the course of treatment. Local lesion care (oral prophylaxis, anesthetic and astringent mouthwash) and management of secondary bacterial infection were additionally carried out. Patient responded well to the treatment with regression of lesions (Figure 6). Patient did not turn up for prolonged follow-up visits so complete (100%) degree of cure could not be assessed.

## 3. Discussion

Leishmaniasis is an infection by protozoans of the genus* Leishmania*. Early descriptions of the leishmanial parasite in cutaneous lesions were done by Cunningham, Borovsky, and Wright between 1885 and 1903 [6]. Other forms of leishmaniasis were described later. In 1903, Leishman and Donovan separately described a protozoan parasite found in the splenic tissue of patients in India [7, 8]. Their simultaneous discovery of the protozoan now called* Leishmania donovani* first alerted the scientific community to the life threatening disease of visceral leishmaniasis. This illness was included by the World Health Organisation in the list of neglected tropical diseases targeted for elimination by 2015 [9].

Now a century later, millions are still afflicted by Leishmania. It is a disease known for its complexity and diversity. It is endemic, in regions ranging from the rainforests of South America to the deserts of Asia, and afflicts both rural and urban communities. A host of about 21 different species of Leishmaniasis is classified under its primary syndromes:* cutaneous, mucocutaneous, and visceral,* which result from parasite multiplication in macrophages in the skin, nasal-oral mucosa, and internal organs, respectively. These protozoan species are transmitted by over 30 species of phlebotomine sand flies.

Clinical manifestation of leishmaniasis depends on the interaction between the characteristic virulence of the species and the host immune response [10]. Mucosal leishmaniasis is a chronic infection of the mucosal membranes, which in most cases is primary but may develop during or after an attack of visceral leishmaniasis. There are at least 30 species of Leishmania, of which 12 named and several unnamed species affect man [11].

### 3.1. Pathogenesis

Leishmania lives two quiet separate lives—one in the sandfly and the other in mammals. In the sandfly, the organism exists as the promastigote (leptomonad), and in tissue it exists as the amastigote (leishmanial or aflagellar form) [12]. The vector sucks the blood of humans or mammals, containing the protozoan in its amastigote form (lacking a flagellum). Once within the intestine of the vector, the parasite transforms into a promastigote (equipped with a flagellum) and reproduces as such. From the intestine of the vector, the protozoan then migrates to the esophagus, where it is expelled into the skin of the host when the insect bites the host, followed by invasion of the bloodstream and different tissues. The promastigotes are phagocytosed by the macrophages of the host reticuloendothelial system, where they lose the flagellum and once again transform into amastigotes [1, 4]. When the infected cells are destroyed, the parasite infects new cells and thus spreads throughout the body. Histological picture of leishmaniasis consists of epithelium with chronic inflammatory infiltrate mainly composed of plasma cells and histiocytes, with cytoplasm invaded by Leishmania parasites.

Leishmaniasis has been reported from different parts of the world and in almost all cases involves the oral mucosa [13–15]. A case has been reported in literature where recurrent mucosal leishmaniasis was associated with Good syndrome (characterized by immunodeficiency in patients with thymoma) and was treated by multiple treatment regimens. Immunodeficiency needs to be highly suspected in patients suffering from recurrent leishmaniasis [16, 17].

The World Health Organization (WHO) estimates that 2-3% of AIDS patients have developed leishmaniasis as opportunistic infection [1]. Mucosal leishmaniasis caused by* Leishmania braziliensis* is known to affect 1–10% of cases, generally developing 1–5 years after the healing of cutaneous leishmaniasis, though in some cases mucosal and skin lesions may occur simultaneously [5]. It is the most feared form of cutaneous leishmaniasis because it produces destructive and disfiguring lesions of the face. It is more common in workers, travelers and visitors to rural and forested areas in countries or areas at risk.

### 3.2. Treatment

The treatment of choice in all clinical forms of leishmaniasis is the administration of antimonial drugs such as meglumine antimoniate (Glucantime) and sodium stibogluconate (Pentostam). Meglumine is given in doses of 20 mg/kg b.w./day, via the intramuscular or intravenous route, to a maximum of 850 mg/day, for at least 20 days. The possible adverse effects of these drugs are myalgia, joint pain, anorexia, nausea, vomiting, headache, hypertransaminasemia, chemical pancreatitis, thrombocytopenia, and neutropenia.* Sodium stibogluconate *(*Pentostam*) dosage recommendations are based on the findings of the WHO Expert Committee on leishmaniasis which met in 2010. 

*Visceral Leishmaniasis*. 20 mg pentavalent antimony (0.2 mL Pentostam) per kg bodyweight daily is given intramuscularly or intravenously for 30 days (or 28 days for* L. infantum*). Patients should be examined for evidence of relapse after 2 and 6 months.

*Cutaneous Leishmaniasis Caused by Old World Species*. In certain cases of Old World CL (OWCL), lesions have seen to spontaneously heal without any need for therapeutic intervention. Local therapies (thermotherapy, cryotherapy, paromomycin ointment, and local infiltration with antimonials) are good options with less systemic toxicity, reserving systemic treatments (azole drugs, miltefosine, antimonials, and amphotericin B formulations) mainly for complex cases.

 
*For lesions requiring local therapy*: 100–500 mg intralesional pentavalent antimony (1–5 mL sodium stibogluconate injection) per session every 3–7 days for 1–5 sessions.
 
*For lesions requiring systemic therapy*: 20 mg/kg pentavalent antimony (0.2 mL sodium stibogluconate injection) intramuscularly or intravenously for 10–20 days.

Monotherapy with sodium stibogluconate is not recommended for the treatment of cutaneous leishmaniasis caused by* L. aethiopica*.

*Cutaneous Leishmaniasis Caused by New World Species*. The majority of New World CL (NWCL) cases require systemic treatment (mainly with pentavalent antimonials), either to speed healing or to prevent dissemination to oral-nasal mucosa as mucocutaneous leishmaniasis [18].

 
*For lesions requiring local therapy*: 100 mg–500 mg intralesional pentavalent antimony (1–5 mL sodium stibogluconate injection) per session every 3–7 days for 1–5 sessions.
 
*For lesions requiring systemic therapy*: 20 mg/kg pentavalent antimony (0.2 mL sodium stibogluconate injection) intramuscularly or intravenously for 20 days.

*Mucocutaneous Leishmaniasis*. Patients should be treated with 20 mg pentavalent antimony (0.2 mL Pentostam) per kg bodyweight daily intramuscularly or intravenously for 30 days and intralesional injection of sodium stibogluconate (0.5 to 1.0 mL) can be effective. It offers less expensive alternative and low side effects [19].

Alternative treatment drugs are amphotericin B, pentamidine, rifampicin, or ketoconazole. Other drugs that have been used for treatment are phenothiazines, paromomycin, allopurinol, itraconazole, cyclosporine, miltefosine, dapsone, aminosidine, and interferon. Additional management options include surgery, topical treatments, immunotherapy, and infrared heat [5, 20, 21].

Genetic studies have been conducted for leishmaniasis that include comparing genetic polymorphism of* Leishmania* (*Viannia*)* braziliensis* in cases of mucosal leishmaniasis from HIV-infected and non- HIV-infected patients. Results have shown genetically divergent profiles in the cutaneous and mucosal lesions of the same HIV- infected patient [22].

Differential diagnosis of oral mucosal leishmaniasis must be established with fungal infections (blastomycosis), syphilis, tuberculosis, leprosy, sarcoidosis, midline granuloma, Wegener's disease, and neoplasms, among other disorders [23, 24].

## 4. Conclusion

Most common form of leishmaniasis is cutaneous leishmaniasis; however exclusive involvement of oral mucosa is rare as in our case. Localized oral mucosal leishmaniasis is an uncommon event in an immunocompetent patient. Dentists play an important role in the diagnosis of oral leishmaniasis, which has systemic infestations. Diagnosing oral leishmaniasis presents a challenge but must be considered in differential diagnosis of exophytic lesions of oral mucosa. Early diagnosis is necessary to provide prompt treatment and to avoid recurrences.
